# Identification of testis development-related genes by combining Iso-Seq and RNA-Seq in *Zeugodacus tau*

**DOI:** 10.3389/fcell.2024.1356151

**Published:** 2024-03-11

**Authors:** Peipei Liu, Ziniu Li, Qiuyuan Zhang, Jiao Qiao, Chenjun Zheng, Wenping Zheng, Hongyu Zhang

**Affiliations:** ^1^ National Key Laboratory for Germplasm Innovation & Utilization of Horticultural Crops, Huazhong Agricultural University, Wuhan, Hubei, China; ^2^ Hubei Hongshan Laboratory, Huazhong Agricultural University, Wuhan, Hubei, China; ^3^ China-Australia Joint Research Centre for Horticultural and Urban Pests, Huazhong Agricultural University, Wuhan, Hubei, China; ^4^ Institute of Urban and Horticultural Entomology, Huazhong Agricultural University, Wuhan, Hubei, China; ^5^ College of Plant Science and Technology, Huazhong Agricultural University, Wuhan, Hubei, China

**Keywords:** *Zeugodacus tau*, Iso-Seq, RNA-Seq, testis development, spermatogenesis, vitamin metabolism

## Abstract

**Introduction:** *Zeugodacus tau* (Walker) is an invasive pest. An effective method to control this pest is the sterile insect technique (SIT). To better apply this technique, it is necessary to understand testis development progression.

**Methods:** Differentially expressed genes (DEGs) during testis development were analyzed by PacBio Iso-Seq and RNA-seq.

**Results:** RNA-Seq library of *Z. tau* testes on day 1, 6, and 11 post eclosion were constructed. We identified 755 and 865 differentially expressed genes in the comparisons of T6 (testes on day 6) vs. T1 and T11 vs. T1, respectively. The KEGG pathway analysis showed that the DEGs were significantly enriched in retinol metabolism, vitamin B6 metabolism, and ascorbate and aldarate metabolism pathways. Knockdown of *retinol dehydrogenase 12*-like (*rdh12*-like), *pyridoxal kinase (pdxk)* and *regucalcin (rgn)*, the representative gene in each of the above 3 pathways, reduced the hatching rate of *Z. tau* offspring. In addition, we identified 107 *Drosophila* spermatogenesis-related orthologous genes in *Z. tau*, of which *innexin 2 (inx2)* exhibited significantly up-regulated expression throughout testis development, and the knockdown of this gene reduced offspring hatching rate.

**Discussion:** Our data indicated that *rdh12*-like, *pdxk*, *rgn*, and *inx2* genes were related to testis development, and they were conserved in tephritid species. These results suggested that this gene might have the same function in tephritid. The findings provide an insight into testis development and spermatogenesis in tephritid species.

## Introduction

*Zeugodacus tau* (*Z. tau*) is one of the most destructive members of tephritid family, and it is also an internationally quarantined pest (Li et al., 2020; He et al., 2023). Female *Z. tau* lays its eggs under the epidermis of vegetables and fruits, causing irreversible damage, thus resulting in huge economic loss (Singh et al., 2010). For example, the potential economic loss caused by *Z. tau* in the pumpkin industry has been estimated to be 37.42–23 157.83 million Yuan per year in China (Fang et al., 2015). Furthermore, *Z. tau* has a wide host range including Anacardiaceae, Cucurbitaceae, and Fabaceae families. Currently, common methods for controlling tephritid fruit flies include chemical control, biological control, and physical control (such as pheromone trapping and yellow board attracting), of which chemical insecticides are considered to be the most effective method (Dias et al., 2018; Garcia et al., 2020). However, chemical insecticides can induce resistance in insects, and they do not effectively kill larvae in infested fruits and vegetables (Baronio et al., 2019). Recently, genetic-based control techniques such as the sterile insect technique (SIT) exhibit great potential for fruit fly management (Pérez-Staples et al., 2020).

The fertility of male insects is important to population breeding. The main strategy of SIT is to release sterile males into the environment, and SIT has already been used to control insects of medical importance and agricultural pests (Harris et al., 2012; Manrakhan and Addison, 2014). The traditional SIT technique uses irradiation or chemicals to induce male sterility. One of the major causes of irradiation infertility is radiation-induced spermatogenic dysfunction, thus further causing the lack of sperm. For example, in *Plutella xylostella*, 400 Gy irradiation causes the damage to the mitochondria and DNA in the testis tissue, in turn causing cell autophagy and apoptosis, further affecting the normal life activities of sperm cells, eventually greatly weakening sperm motility and insemination ability (Li et al., 2023). However, irradiation affects the effectiveness of pest control by reducing the mating ability of male insects. In addition to radiological or chemical methods, some other methods can also cause abnormal testis development and male sterility. For example, in *Anastrepha suspensa*, the injection of dsRNA of *transformer* and *transformer-2* into fertilized embryos leads to XX males. These XX phenotypic males are infertile due to hypertrophic testes and reduced sperm motility (Schetelig et al., 2012). In *Bactrocera dorsalis*, RNAi of embryonic *typo-gyf* causes abnormal testis development and a significant reduction in sperm number, thus resulting in a lower hatching rate of offspring (Liu et al., 2024). Overall, understanding testicular development progression is critical for successful use of SIT. Testicular development is a complex physiological processes including cell cycle progression, spermatogenesis, sperm formation, and others (Li et al., 2009; Lie et al., 2009; Dong et al., 2015). Testicular development involves a large number of tissue-specific genes and proteins because the spermatid exhibits the highest differentiation degree among all cell types (Djureinovic et al., 2014). Many studies have been carried out to explore the genes regulating testis development, especially in mammals. The testicle appears to have a higher metabolic activity relative to other normal tissues. Ramsköld et al. (2009) evaluated multiple tissues from mammals by RNA-Seq and found that many genes were specifically expressed in testicular samples (Ramsköld et al., 2009). Djureinovic et al. (2014) categorized 20,050 putative human genes based on expression patterns and found testis had the largest quantity of tissue-specific genes compared to other tissues (Djureinovic et al., 2014). Several studies have investigated testicular development in insects. For example, the transcriptomes of the male reproductive organs in *Lutzomyia longipalpis* (Azevedo et al., 2012), *Aedes aegypti* (Akbari et al., 2013; Degner et al., 2019), and *B. dorsalis* (Wei et al., 2014; Tian et al., 2017) have been sequenced, and thousands of new genes related to male reproductive development have been identified. *Z. tau* males sexually mature from day 8 after emergence and begin to mate. Over time, the mating rate gradually increases, and it reaches up to 70% on the 11th day (our unpublished data). The longer sex development period of *Z. tau* male adult than *B. dorsalis*is suggests the more complicated reproductive development process of *Z. tau* male. However, there are few studies on the male reproductive development of *Z. tau*. Considering this, the current study selected *Z. tau* as research subject.

High-throughput sequencing based on the Illumina platform is an effective method for gene annotation and expression quantification. However, the reads produced by RNA-Seq technology are relatively short with an average read length of only 100-150 bp, and they usually cannot cover the entire transcript, thus reducing the accuracy of sequence assembly (Liu et al., 2012). The PacBio Iso-Seq (single-molecule long-read isoform sequencing) is a recently developed third-generation sequencing technology. Iso-Seq is characterized by long reads, and it can detect base modification. The average length of reads generated by Iso-Seq reaches 10-15 kb, which can meet the requirements for obtaining full-length transcripts (Roberts et al., 2013; Wang et al., 2019). Iso-Seq has multiple advantages over other sequencing methods such as higher resolution, higher sensitivity, better capability to identify new transcript sequences, alternative splicing events, variable polyadenylation events, and fusion genes. Iso-Seq readings were aligned with RNA-seq data using the tool LoRDEC to validate sequence accuracy. The combination strategy of Iso-Seq and RNA-Seq allows the generation of correct transcripts and high-quality profiles of genes and the quantification of transcript expression (Jiang et al., 2017). The combined strategy has been used to identify and quantify full-length transcripts in several species, including *Tuta absoluta*, *Picromerus lewisi*, *Rhynchophorus ferrugineus*, and *Anopheles stephens* (Jiang et al., 2017; Yang et al., 2020; Li et al., 2022; Liu et al., 2023). However, this combined strategy has not been reported in *Z. tau.*

The aim of this study is to explore the change in testis development in *Z. tau* at the transcriptional level. First, the comprehensive transcriptome profiles of *Z. tau* testes in different developing stages were obtained by combining Iso-Seq and RNA-Seq. Second, KEGG pathway enrichment analysis indicated that vitamin metabolism pathways were significantly enriched by differentially expressed genes (DEGs). Third, the orthologous genes associated with *Drosophila* spermatogenesis were identified from the testicular transcriptome database of *Z. tau*. Collectively, our study reveals a potential molecular mechanism underlying male reproduction in *Z. tau*, and our findings enrich gene resources for SIT.

## Materials and methods

### Insect rearing

*Z. tau* used in this study was reared at the Institute of Horticultural and Urban Entomology at Huazhong Agricultural University (Wuhan, China). The larvae were reared on an artificial diet containing pumpkin, corn, yeast powder, wheat bran, and sucrose. The adults were reared on an artificial diet containing 50% yeast powder and 50% sucrose. *Z. tau* at various life stages was cultured at 27°C ± 1°C under a 12 light: 12 dark photoperiod in cages (Liu et al., 2021).

### Imaging

Newly emerged adults were collected and sexed within 12 h after emergence, and males were subsequently maintained in separate cages. The day of emergence was defined as the first day. Males were dissected in 1X PBS solution on day 1, 6, and 11 after eclosion, and testes were washed two times with PBS. Images were captured under Nikon C-PS stereo microscope equipped with a Nikon digital camera D5100 (Nikon, Japan). Testis length and width were measured using ImageJ software.

### Sample preparation and RNA extraction

Testis tissues from male adults of *Z. tau* were dissected in 1X PBS solution on day 1, 6, and 11 after eclosion and snap-frozen in liquid nitrogen. Each sample had three biological replicates with 30 pair of testes per replicate. Total RNA was extracted from testes using RNAiso Plus reagent (TaKaRa, Japan) according to the manufacturer’s protocol and quantified using a NanoDrop 2000c spectrophotometer (Thermo Fisher Scientific, United States).

### Library preparation and sequencing

Library was prepared and sequenced by Novogene (Novogene Co., Ltd.). The 1.5 µg RNA from the testis samples was prepared and RNA-Seq library was performed using a NEBNext^®^ Ultra™ RNA Library Prep Kit for Illumina^®^ (NEB, United States). The first-strand cDNA was synthesized using M-MuLV Reverse Transcriptase and random hexamer primers. The second-strand cDNA was synthesized using buffer, dNTPs, DNA Polymerase I, and RNase H. The library fragments were purified on an AMPure XP system (Beckman Coulter, United States) to obtain cDNA fragments with lengths of 250–300 bp. The library was prepared and subjected to RNA-Seq on an Illumina HiSeq 6000 platform using 150 bp paired-end technology.

The equal amount of the total RNA samples from all three testis developing stages were pooled together and used as the template for cDNA. The Iso-Seq libraries were constructed with the SMARTer PCR cDNA Synthesis Kit (Clontech, United States) and BluePippin Size-Selection System (Sage Science, United States). The Iso-Seq libraries were sequenced using PacBio Sequel II system.

### Analysis of sequencing data

The RNA-Seq raw reads were filtered to remove adaptor sequences, ambiguous reads with ‘N’ bases, and low-quality reads. The Iso-Seq raw reads were processed using the CCS v6.2.0 software to obtain Circular Consensus Sequences (CCSs). Then the CCSs were subsequently classified into full-length non-chimeric sequences (FLNC) (as defined by the presence of 5′ primer, 3′ primer, and the polyA tail if applicable) and non-full-length sequences (NFL). Lima v2.1.0 and isoseq3 refine were used to remove the primers and poly(A) tails, respectively. Subsequently, the clustering algorithm ICE was used to obtain high-quality FL consensus sequences. Subsequently, high-quality FL consensus sequences were mapped to the *Zeugodacus cucurbitae* reference genome (GCA_028554725.2) with the Minimap2 program, and the redundant transcripts removed by cDNA_Cupcake 28.0.0.

### Functional annotation of transcripts

Transcript sequences were annotated based on the following five databases: the KEGG Ortholog database (KEGG), evolutionary genealogy of genes:Non-supervised Orthologous (eggNOG), Gene Ontology (GO), NCBI non-redundant protein sequences (NR), and Translation of EMBL (TrEMBL).

### Gene structure analysis

Alternative splicing (AS) including mutually exclusive exon (MEE), intron retention (IR), exon skipping (ES), alternative 3ʼ splice site (A3), and alternative 5ʼ splice site (A5) were identified by the AStalavista tool. Alternative polyadenylation (APA) site detection was performed using TAPIS pipeline (TAPIS 1.1.3). Transcription factors (TFs) were identified and assigned into different families based on animal transcription factor database (AnimalTFDB 3.0). Furthermore, the TransDecoder software was used to identify reliable potential CDSs from transcript sequences based on open reading frame (ORF) length, log-likelihood score, and alignment between amino acid sequence and protein domain sequences in Pfam database.

### LncRNA and simple repeat sequences identification

Transcripts were screened for putative lncRNAs using four tools, including coding-non-coding index (CNCI), coding potential calculator (CPC), coding potential assessment tool (CPAT), and Pfam database. The putative lncRNAs were further screened using the intersection CPC, CNCI, CPAT and Pfam results. Simple repeat sequences (SSRs) were identified by MISA software (MIcroSAtellite identification tool).

### *Drosophila* spermatogenesis orthologous gene search

*Drosophila* spermatogenesis protein names from the Uniport database (https://www.uniprot.org/) were used to search the orthologous genes against *Z. tau* testis transcriptome database by tBLASTn.

### Differentially expressed genes

The gene expression levels were calculated as fragments per kilobase of transcript per million fragments mapped (FPKM). The DEGs were identified using the differentially expressed sequencing (DESeq2) method based on the raw count data. The DEGs were identified with the thresholds of padj-value <0.05 and |log2 FC (fold change)| > 1. The FPKM values were normalized with log2 transformation and used to generate the hierarchical clustering with the pheatmap R package. Then the KEGG enrichment analysis of DEGs was performed using KOBAS software.

### Selection of target genes

In the retinol metabolism pathway, retinoic acid (RA) is the main metabolite of vitamin A in the body. RA is synthesized from an inactive precursor through a two-step enzymatic oxidation reaction in which retinol is first converted into retinal then into RA. The RDH family is involved in the first-step reaction, and the expression of *rdh12*-like increases significantly with testicular development, suggesting the potential role of this gene in testicular development. In the vitamin B6 metabolic pathway, *pdxk* and *pnpo* are involved in the synthesis of pyridoxal 5′-phosphate (PLP) related to the spermatogenesis process. The analysis of differential genes showed that the fold change of *pdxk* was slightly larger than that of *pnpo,* suggesting that *pdxk* might be more related to spermatogenesis. In the ascorbate and aldarate metabolism pathway, L-gulono-1,4-lactone and L-galactono-1,4-lactone are the precursors of L-Ascorbate, their synthesis is regulated by the RGN and GalDH families, and the expression level of *rgn* (LOC105209294) in the testes is significantly higher than that of LOC105209295, LOC105210018, and LOC105218123 (the other 3 genes in these two gene families). The analysis of 107 *Drosophila* homologous gene reveals that only the expression of *inx2* changes significantly in the development of the testes. Therefore, we selected the above-mentioned 4 genes (*rdh12*-like, *pdxk*, *rgn*, and *inx2*) for subsequent functional analysis.

### Quantitative real-time PCR

Quantitative real-time PCR (qPCR) was performed to validate the expression of the target genes obtained by RNA-Seq. Total RNA was extracted from T1, T6, and T11 (testes on day 1, 6, and 11 post eclosion), and used as template for first-strand cDNA synthesis with reverse transcriptase. The qPCR was performed in a 10 μL reaction system containing 2 μL of cDNA, 0.4 μL of each primer (10 mM), 5 μL of mix (Hieff UNICON^®^ qPCR SYBR Green Master Mix, YEASEN), and 2.2 μL of ddH_2_O. The qPCR program was as follows: predenaturation at 95°C for 5 min, then 40 cycles of denaturation at 95°C for 15 s, annealing and extension at 60°C for 30 s. To ensure PCR product specificity, the melting curve for all the reactions was analyzed from 60°C to 95°C. The primers used in this study were presented in Supplementary Table S1. The ribosomal protein gene *rpl32* was used as an internal control. After normalization using the control gene, the expression levels of target genes during testis development were determined using the 2^−ΔΔCT^ method (Livak and Schmittgen, 2002).

### RNA interference

The target gene fragments were PCR amplified with cDNA from adult *Z. tau* as the template, and one T7 promoter sequence was introduced to 5′ end of the primer pair. A control *egfp* fragment was amplified with the primer pair reported by Peng et al. (2020). The PCR was performed with 2 × Prime-STAR^®^ Max DNA Polymerase (Takara, Japan). The PCR products were purified with the E. Z.N.A.^®^ Cycle-Pure Kit (Omega, United States). The dsRNAs were synthesized with the T7 RiboMAX™ Express RNAi System (Promega, United States), according to the manufacturer’s instructions. The synthesized dsRNAs were verified through 1.5% agarose gel electrophoresis and quantified with a NanoDrop 2000c spectrophotometer (Thermo Fisher Scientific, United States). The dsRNA was diluted to 2,000 ng/μL and microinjected into male adult (7 days post eclosion).

### Hatching rate of offspring

Virgin females and males were collected, and mating was conducted on day 11 after eclosion. After mating, females and males were separated, and females were fed. On the 2nd day after mating, females were induced to lay eggs by pumpkin piece for 4 h (15:00–19:00). The collected eggs were placed on moist filter paper and maintained at 27°C ± 1°C and 70% ± 5% humidity. There were 50 eggs in each replicate, and the number of eggs hatched in each replicate was counted at hour 48 after laying.

### Statistical analysis

Statistical analyses were conducted using GraphPad Prism 8 (GraphPad Prism Software, United States). Student’s t-test was used to compare the means of two independent samples. One-way ANOVA and Tukey’s HSD test were used for multiple comparisons of multiple samples. *p* < 0.05 was considered statistically significant.

### Phylogenetic analysis

A nucleotide sequence homology analysis was performed based on NCBI, and a phylogenetic tree was constructed with the MEGA 7.0.26 software using the maximum likelihood method. *Drosophila innubila* or *Drosophila melanogaster* was used as outgroup to construct *Phylogenetic* trees.

### Selection analyses

Non-synonymous substitution rate (Ka) and synonymous substitution rate (Ks) were calculated using Tbtools II (v2.039) software.

## Results

### Testes morphology changes with eclosion days

**FIGURE 1:**
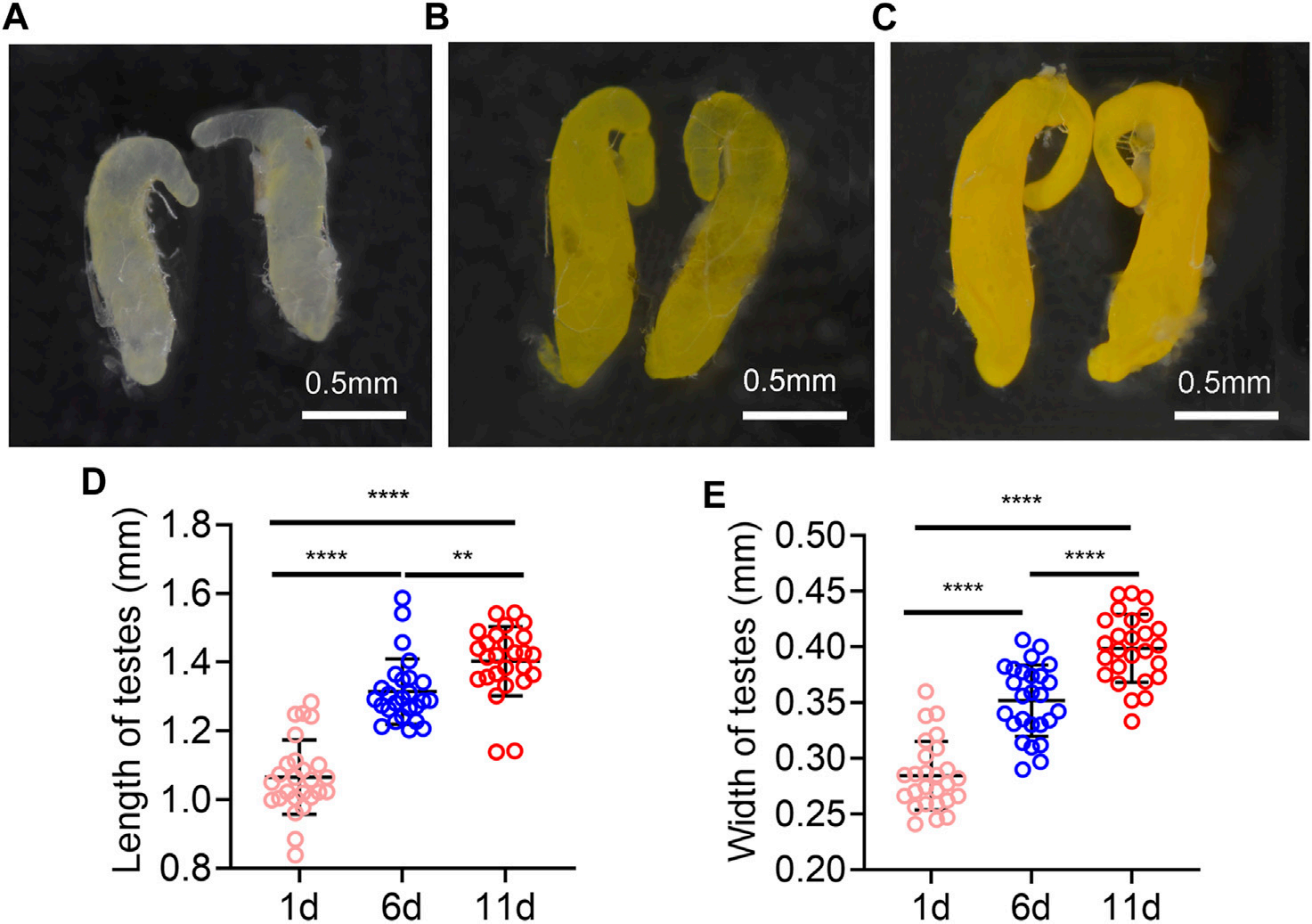
Testes morphology of Z. tau changes with eclosion days. The morphology testes on day 1 **(A)**, 6 **(B)**, and 11 **(C)** post eclosion. The length **(D)** and width **(E)** of testes on day 1, 6, and 11 post eclosion (**, *p* < 0.01; ****, *p* < 0.0001).

The testes on day 1 post eclosion were light yellow, and with the increasing days, the color of the testes was deepened. It turned yellow on day 6 and darker on day 11 (Figures 1A–C). In addition, the length and width of the testes were increased with time extension post eclosion, the testis length and width were significantly longer on day 11 and 6 than on day 1, and they were significantly longer on day 11 than on day 6 (Figures 1D, E).

### Sequencing data analysis

To comprehensively characterize the gene expression dynamics, the transcriptome of developing *Z. tau* testes (T1, T6, T11) was generated from Iso-Seq. After quality filtering, a total of 45.37 Gb clean reads were obtained. After a single molecular self-correction, 374,295 CCSs were obtained, and CCSs were subsequently classified into FLNC and NFL, with a proportion of 91.43% and 8.57%, respectively. Consequently, a total of 342,213 high-quality FLNC were obtained. Then these FLNC sequences were mapped onto the *Z. cucurbitae* reference genome, and the mapping rate was 96.74%. After removing redundant sequences, a total of 29,233 full-length non-redundant sequences were generated (Table 1).

**TABLE 1:** SMRT sequencing statistics.

cDNA size	1–6 K
CCS Number	374,295
Read bases of CCS	879,421,714
Mean read length of CCS	2,349
Mean number of passes	41
Number of full-length non-chimeric reads	342,213
Number of consensus isoforms	67,958
Number of polished high-quality isoforms	67,939
Number of polished low-quality isoforms	19
Number of redundant transcript isoforms	29,233

### Function prediction of new transcripts

Of the 29,233 transcripts, 15,943 were identified as new transcripts. The functions of new transcripts were annotated based on KEGG, eggNOG, GO, NR, and TrEMBL databases. As a result, a total of 14,803 (92.84%) new transcripts were annotated, and the Upset Plot showed that 9,596 transcripts were shared by all five databases (Supplementary Figure S1).

### Transcription factor, alternative splicing and alternative polyadenylation analyses

**FIGURE 2:**
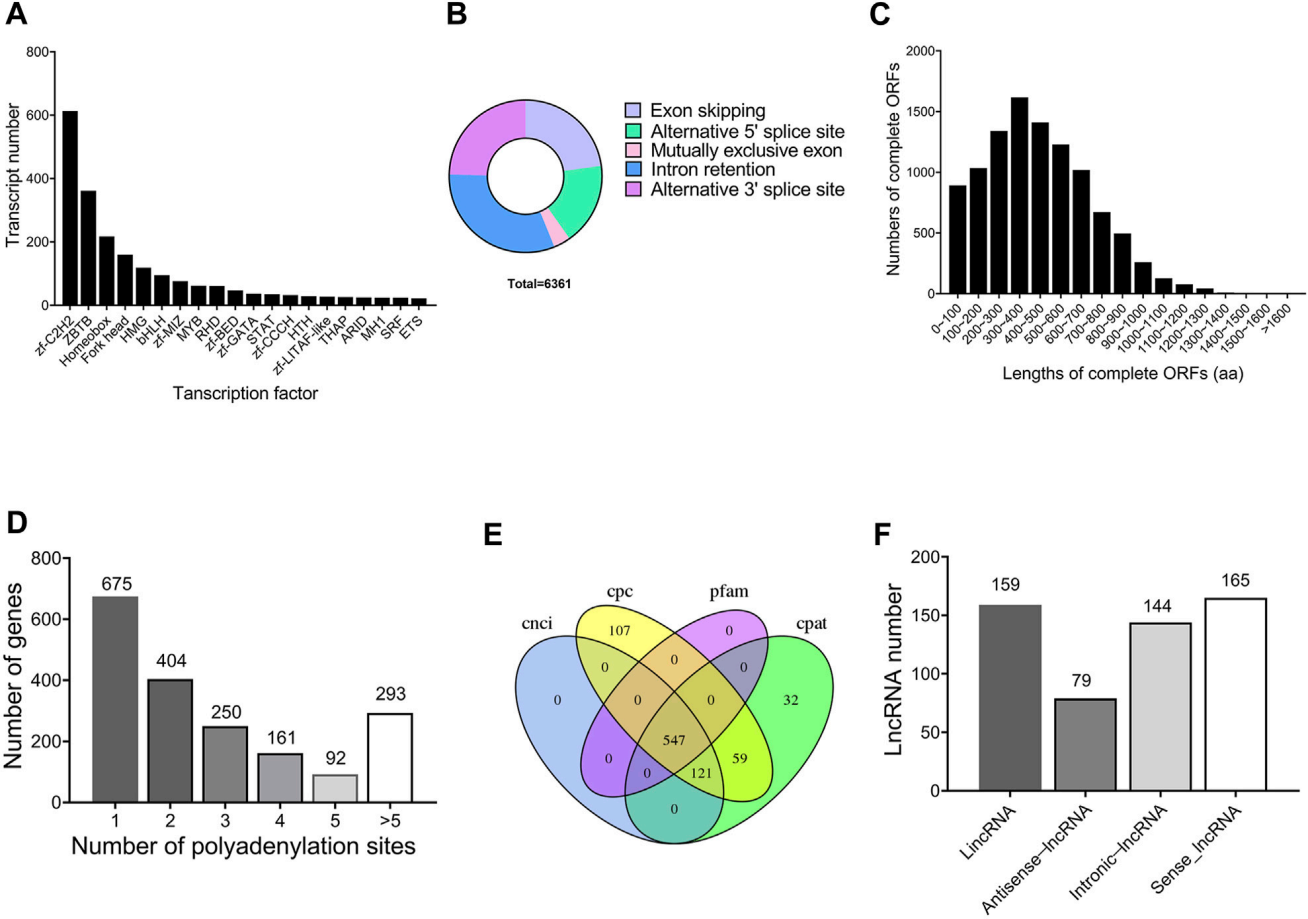
Structure characteristics of genes throughout the testis development of *Z. tau.* **(A)** Main transcription factor (TF) families and number of transcripts contained in the corresponding TF families. **(B)** Five types of alternative splicing (AS) events. **(C)** Number of the complete ORFs with different lengths. **(D)** Distribution of APA sites in the genomic regions. **(E)** Venn diagram of lncRNA number predicted by four methods (CNCI, CPC, PLEK, and Pfam). **(F)** Numbers of four types of lncRNAs (LincRNA, Antisense-lncRNA, Intronic-lncRNA and Sense-lncRNA).

A total of 2,404 transcripts were functionally annotated as transcription factors. The top three families were 613 bZIP (25.50%), 361 ZBTB (15.02%), and 217 Homeobox (9.03%), respectively (Figure 2A). Using Astalavista software, 6,361 AS events were identified, of which 230 (3.62%) AS events were identified as MEE, 2,004 (31.50%) as IR, 1,451 (22.81%) as ES, 1,109 (17.43%) as A5, and 1,567 (24.64%) as A3 (Figure 2B). In addition, 14,783 ORFs were obtained, including 10,241 complete ORFs (Figure 2C). The distributions of the numbers and lengths of complete ORFs were shown in Figure 2A. Further, we identified 675 genes with only one APA site, 404 genes with two APA sites, 250 genes with three APA sites, 161 genes with four APA sites, 92 genes with five APA sites, and 293 genes with more than five APA sites (Figure 2D).

### Identification of lncRNAs and simple repeat sequences

In this study, a total of 547 transcripts were identified as long noncoding RNAs (lncRNAs) by CPC, CNCI, Pfam, and CPAT (Figure 2E). According to the location of lncRNAs in reference genome, 159 (29.1%) lncRNAs were identified as lincRNA, 79 (14.4%) lncRNAs as antisense-lncRNA, 144 (26.3%) lncRNAs as intronic-lncRNA, and 165 (30.2%) lncRNAs as sense-lncRNA (Figure 2F). Further, 29,152 new transcripts with length more than 500 bp were analyzed, from which 8,290 new transcripts were identified to contain SSR sequences. From these 8,290 SSR-containing new transcripts, a total of 12,967 simple sequence repeats (SSRs) were identified. Among the 12,967 SSRs, tri-nucleotide SSRs (7,421) were the most abundant, followed by mono-nucleotide SSRs (3,717), and di-nucleotide SSRs (1,658).

### Identification of DEGs during testis development

**FIGURE 3:**
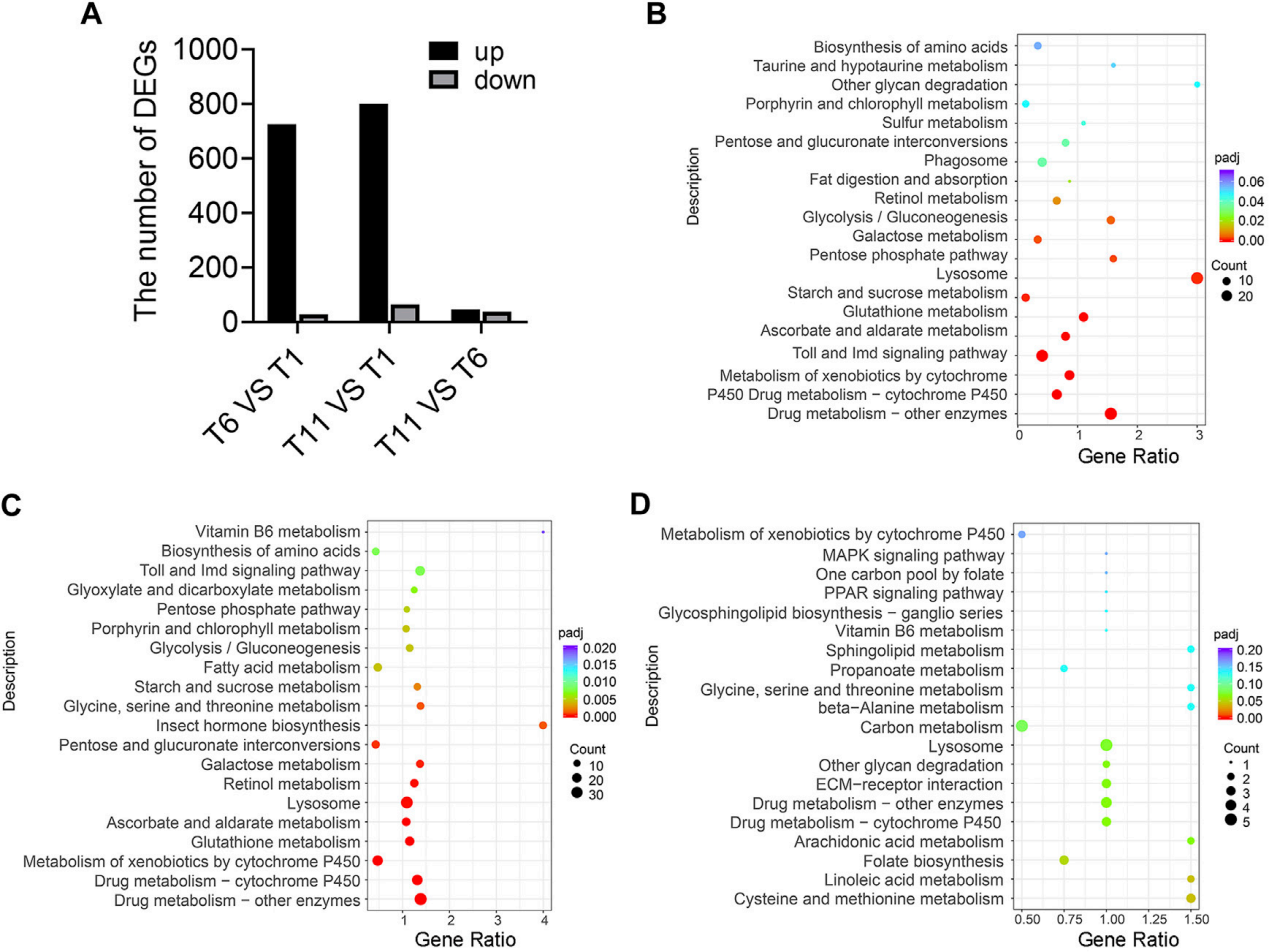
Differentially expressed genes (DEGs) in the testis development of *Z. tau*. **(A)** Number of DEGs in the comparisons of T6 vs. T1, T11 vs. T1, and T11 vs. T6. T1, T6, and T11 indicate testis on day 1, 6, and 11 after eclosion. **(B)** KEGG analysis of the DEGs in T6 vs. T1. **(C)** KEGG analysis of the DEGs in T11 vs. T1. **(D)** KEGG analysis of the DEGs in T11 vs. T6.

Transcriptome sequencing of 9 samples was performed, and Q30 (percentage of sequences with sequencing error rate lower than 0.1%) reached 92.97%–94.16%, indicating that the reads were of high quality and qualified for analysis of DEGs. Furthermore, DEGs were identified in the comparison of T1 vs. T6, T11 vs. T1, and T11 vs. T6 at the three testis development stages. In the comparison of T6 vs. T1, 755 DEGs were identified, of which 726 DEGs were upregulated, and 29 DEGs were downregulated. In the comparison of T11 vs. T1, 865 DEGs were identified, of which 800 DEGs were upregulated, and 65 DEGs were downregulated. In the comparison of T11 vs. T6, 85 DEGs were identified, of which 39 DEGs were upregulated, and 46 DEGs were downregulated (Figure 3A). KEGG enrichment analysis showed that the DEGs in T6 vs. T1 were most significantly enriched in 3 pathways, namely, “Lysosome pathway”, “Toll and Imd signaling pathway”, and “Drug metabolism - cytochrome P450”; that the DEGs in T11 vs. T1 were most significantly enriched in the 3 pathways including “Lysosome pathway”, “Drug metabolism - cytochrome P450”, and “Carbon metabolism”; and that the DEGs in T11 vs. T6 were significantly enriched in 3 pathways including “Linoleic acid metabolism”, “Cysteine methionine metabolism”, and “Folate biosynthesis”. In addition, multiple vitamin-related pathways were significantly enriched. For example, “Retinol metabolism”, and “Ascorbate and aldarate metabolism” pathways were significantly enriched with the DEGs in T6 vs. T1; “Retinol metabolism”, “Vitamin B6 metabolism”, and “Ascorbate and aldarate metabolism” pathways were significantly enriched with the DEGs in T11 vs. T1; and “Retinol metabolism”, “Vitamin B6 metabolism”, and “Folate biosynthesis” pathways were significantly enriched with the DEGs in T11 vs. T6 (Figures 3B–D). These results suggested that vitamin metabolism might be involved in the development of testis.

### Retinol metabolism were involved in testis development

**FIGURE 4:**
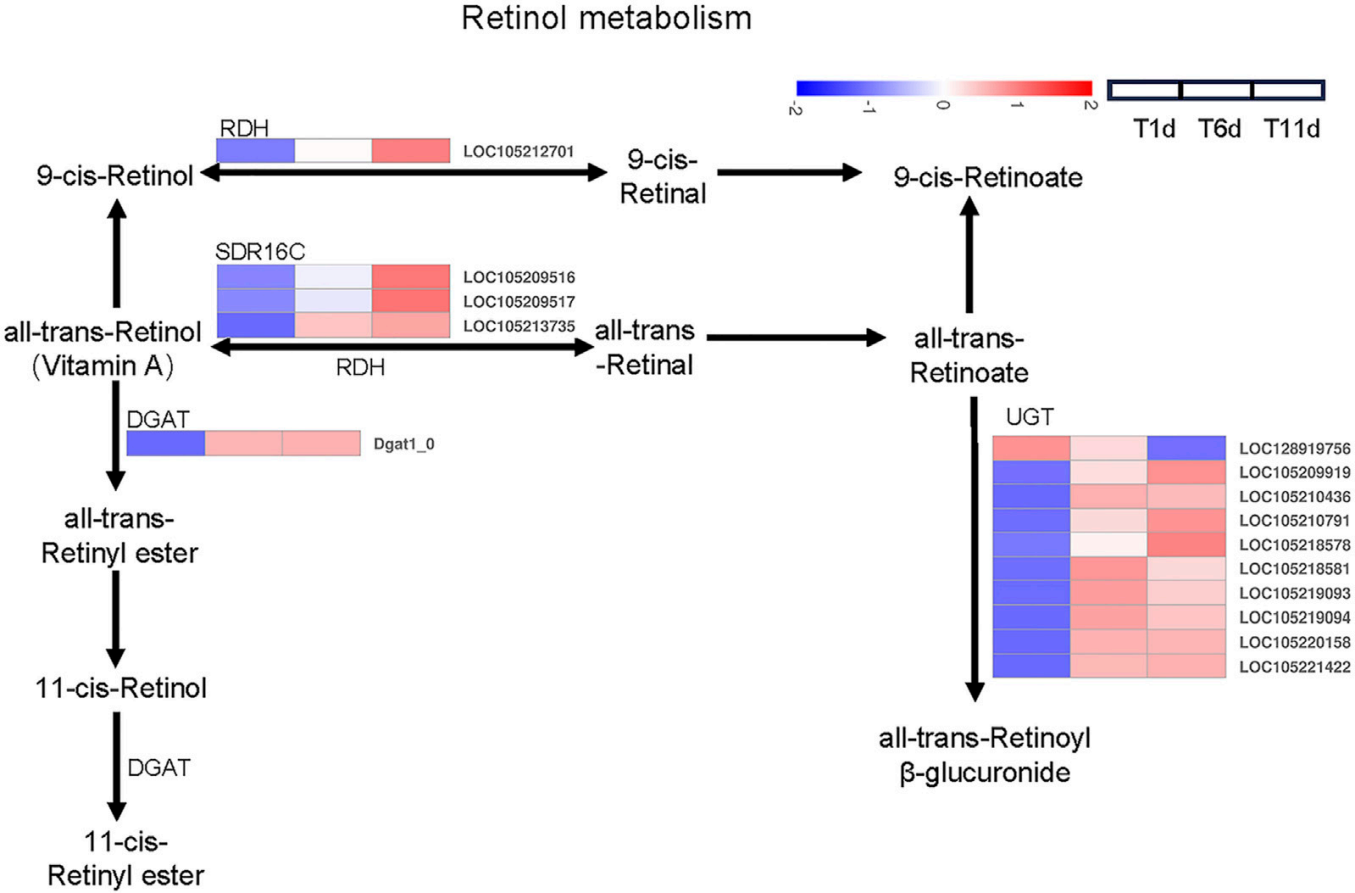
Heatmap of the expression profiles of DEGs involved in retinol metabolism in *Z. tau* testes. T1, T6, and T11 represent testes on day 1, 6, and 11 post eclosion, respectively.

We investigated the DEGs involved in retinol metabolism. Totally, 15 DEGs related to the retinol metabolism were identified across 4 families including 1 gene in retinol dehydrogenase (RDH) family, 3 genes in all-trans-retinol dehydrogenase (SDR16C) family, 1 gene in diacylglycerol O-acyltransferase (DGAT) family, 10 genes glucuronosyltransferase (UGT) family (Figure 4).

Further analysis indicated that among 15 retinol metabolism-related genes, the expression level of 4 genes (2 belonging to SDR16C (LOC105209516 and LOC105213735) and 2 belonging to UGT (LOC105209919 and LOC105220158)) was higher than the remaining 11 genes throughout testis developments (T1–T11). With the development of testis (from T1 to T11), only one UGT gene (LOC128919756) was continuously downregulated. Seven genes (1 RDH (LOC105212701), 3 SDR16C (LOC105209516, LOC105209517, and LOC105213735) and 3 UGT (LOC105209919, LOC105210791, and LOC105218578)) were significantly continuously upregulated. Additionally, the expression level of 1 DGAT (*Dgat1_0*) and 6 UGT (LOC105210436, LOC105218581, LOC105219093, LOC105219094, LOC105220158, and LOC105221422) was significantly increased and plateaued (Table 2). Therefore, these retinol metabolism-associated DEGs with specific expression patterns were highly likely to be involved in testis development.

**TABLE 2:** Differentially expressed genes involved in vitamin metabolism.

Enzyme	Gene ID	T1	T6	T11	T6 VS. T1	T11 VS. T1
Retinol metabolism						
DGAT	Dgat1_0	1.7345	6.761934	6.913999	*	*
RDH	LOC105212701	0.28187	0.682693	1.157319	no	*
SDR16C	LOC105209516	13.39775	34.69321	129.2606	no	*
	LOC105209517	0.256519	0.672628	1.740267	no	*
	LOC105213735	13.17173	51.44456	63.12685	*	*
UGT	LOC128919756	8.726864	5.028652	0.848712	*	*
	LOC105209919	12.46673	21.09953	26.58083	no	*
	LOC105210436	0.416249	1.227206	1.176597	no	*
	LOC105210791	2.563174	6.072815	8.231448	*	*
	LOC105218578	0.473939	3.880796	11.32484	*	*
	LOC105218581	1.9247	4.529353	3.680961	*	*
	LOC105219093	0.553394	2.890756	2.247317	*	*
	LOC105219094	2.26124	14.76726	11.56811	*	*
	LOC105220158	12.73465	27.6289	27.27457	*	*
	LOC105221422	2.08642	5.149338	5.300399	*	*
Vitamin B6 metabolism						
PNPO	LOC105219330	4.915588	10.87719	9.781125	*	*
PDXK	LOC105220897	4.831618	11.59752	11.2213	*	*
PSAT	LOC105211328	12.84718	8.159183	3.695703	no	*
Ascorbate and aldarate metabolism						
UGT	LOC128919756	8.726864	5.028652	0.848712	*	*
	LOC105209919	12.46673	21.09953	26.58083	no	*
	LOC105210436	0.416249	1.227206	1.176597	no	*
	LOC105210791	2.563174	6.072815	8.231448	*	*
	LOC105218578	0.473939	3.880796	11.32484	*	*
	LOC105218581	1.9247	4.529353	3.680961	*	*
	LOC105219093	0.553394	2.890756	2.247317	*	*
	LOC105219094	2.26124	14.76726	11.56811	*	*
	LOC105220158	12.73465	27.6289	27.27457	*	*
	LOC105221422	2.08642	5.149338	5.300399	*	*
MIOX	LOC105208715	142.0905	140.6681	293.9707	*	*
ALDH	LOC128919741	2.394816	20.03496	34.45948	no	*
RGN	LOC105209294	18.66791	187.6682	221.4827	*	*
	LOC105209295	0.21618	1.777363	1.566675	*	*
	LOC105210018	1.452023	13.65683	19.69109	*	*
GalDH	LOC105218123	31.90837	97.9338	70.5362	*	*

Note: * represents significant difference at *p* < 0.05.

### Vitamin B6 metabolism were involved in testis development

**FIGURE 5:**
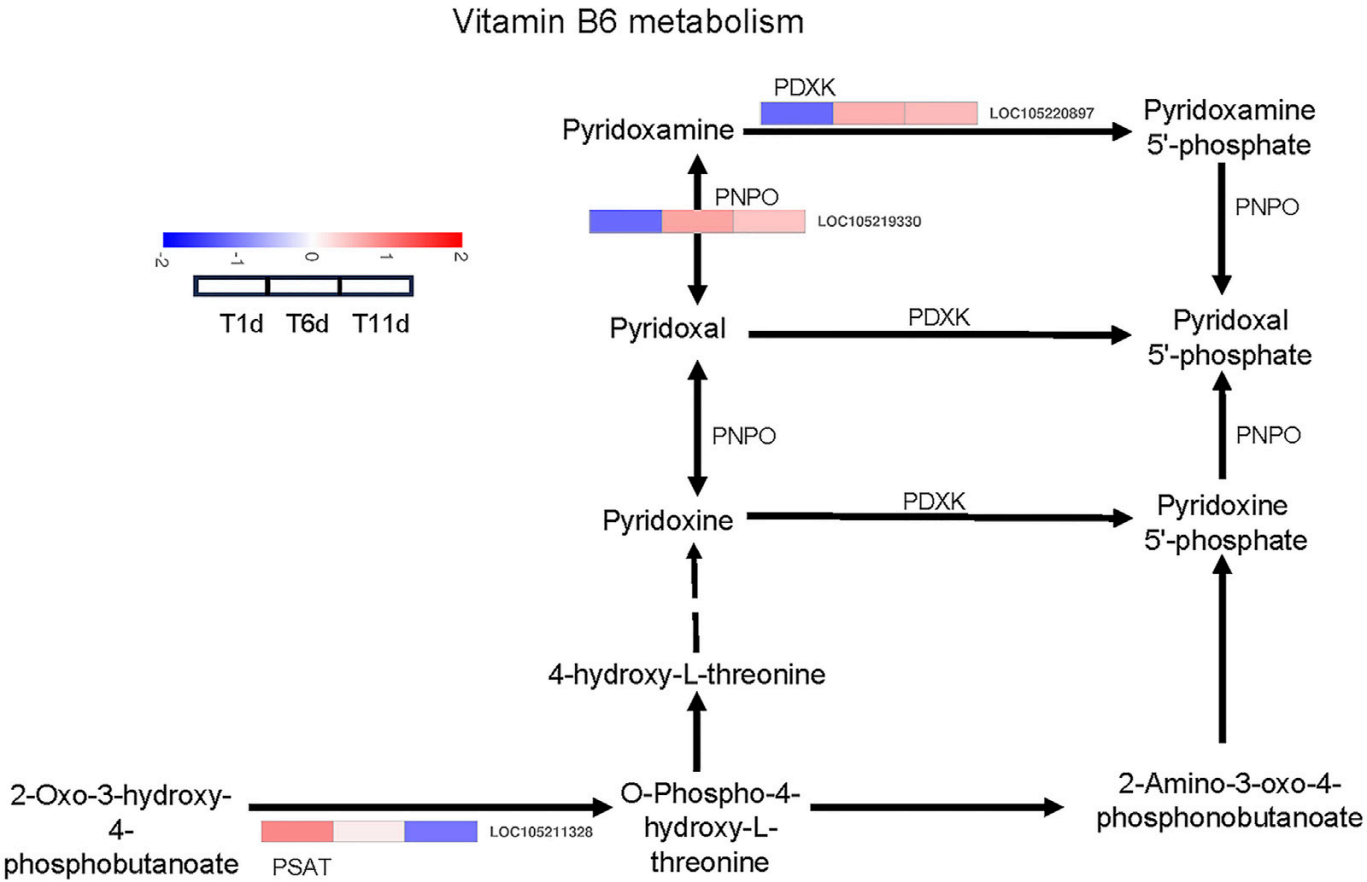
Heatmap of the expression profiles of DEGs involved in vitamin B6 metabolism in *Z. tau* testes. T1, T6, and T11 represent testes on day 1, 6, and 11 post eclosion, respectively.

We investigated the DEGs that encode enzymes involved in vitamin B6 metabolism pathways. As a result, 3 DEGs across 3 families were identified as enzyme-coding genes responsible for vitamin B6 metabolism, including one pyridoxal kinase (PDXK) gene, one pyridoxamine 5′-phosphate oxidase (PNPO) gene, and one phosphoserine aminotransferase (PSAT) gene (Figure 5).

With the development of testis from T1 to T11, the expression level of one PDXK (LOC105220897) and one PNPO (LOC105219330) was significantly increased and plateaued. Additionally, one PSAT gene (LOC105211328) was continuously downregulated (Table 2). Therefore, these vitamin B6 metabolism-associated DEGs with specific expression patterns were highly likely to be involved in testis development.

### Ascorbate and aldarate metabolism were involved in testis development

**FIGURE 6:**
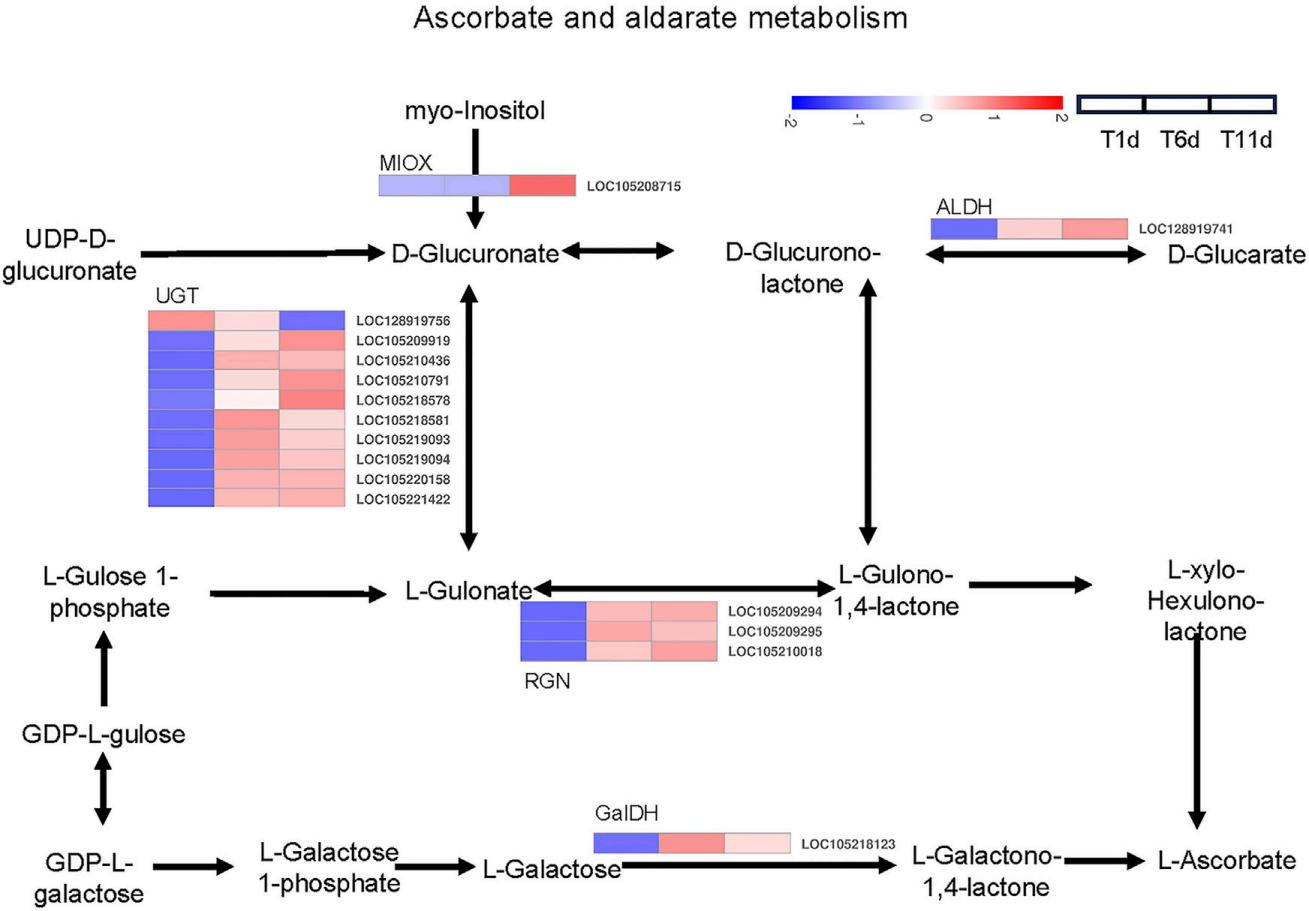
Heatmap of the expression profiles of DEGs involved in ascorbate and aldarate metabolism in *Z. tau* testes. T1, T6, and T11 represent testes on day 1, 6, and 11 post eclosion, respectively.

Based on the KEGG pathway database, a total of 16 DEGs across 5 families in the three testis development stages (T1-T11) were identified to be involved in ascorbate and aldarate metabolism, including 1 inositol oxygenase (MIOX) gene, 10 glucuronosyltransferase (UGT) genes, 1 aldehyde dehydrogenase (ALDH) gene, 3 gluconolactonase (RGN) genes, and 1 L-galactose dehydrogenase (GalDH) gene (Figure 6).

The expression levels of 8 genes including 6 UGT (LOC105210436, LOC105218581, LOC105219093, LOC105219094, LOC105220158 and LOC105221422), 1 RGN (LOC105209295), and 1 GalDH (LOC105218123) were significantly higher in T6 an T11 than in T1, but no significant differences were observed between T11 and T6. With the development of testis from T1 to T11, 7 genes (1 MIOX (LOC105208715), 3 UGT (LOC105209919, LOC105210791 and LOC105218578), 1 ALDH (LOC128919741) and 2 RGN (LOC105209294 and LOC105210018)) were significantly continuously upregulated, whereas only 1 UGT gene (LOC128919756) was continuously downregulated (Table 2). Therefore, these ascorbate and aldarate metabolism-associated DEGs with specific expression patterns were highly likely to participate in testis development.

### Identification of orthologous genes associated with *Drosophila* spermatogenesis

**FIGURE 7:**
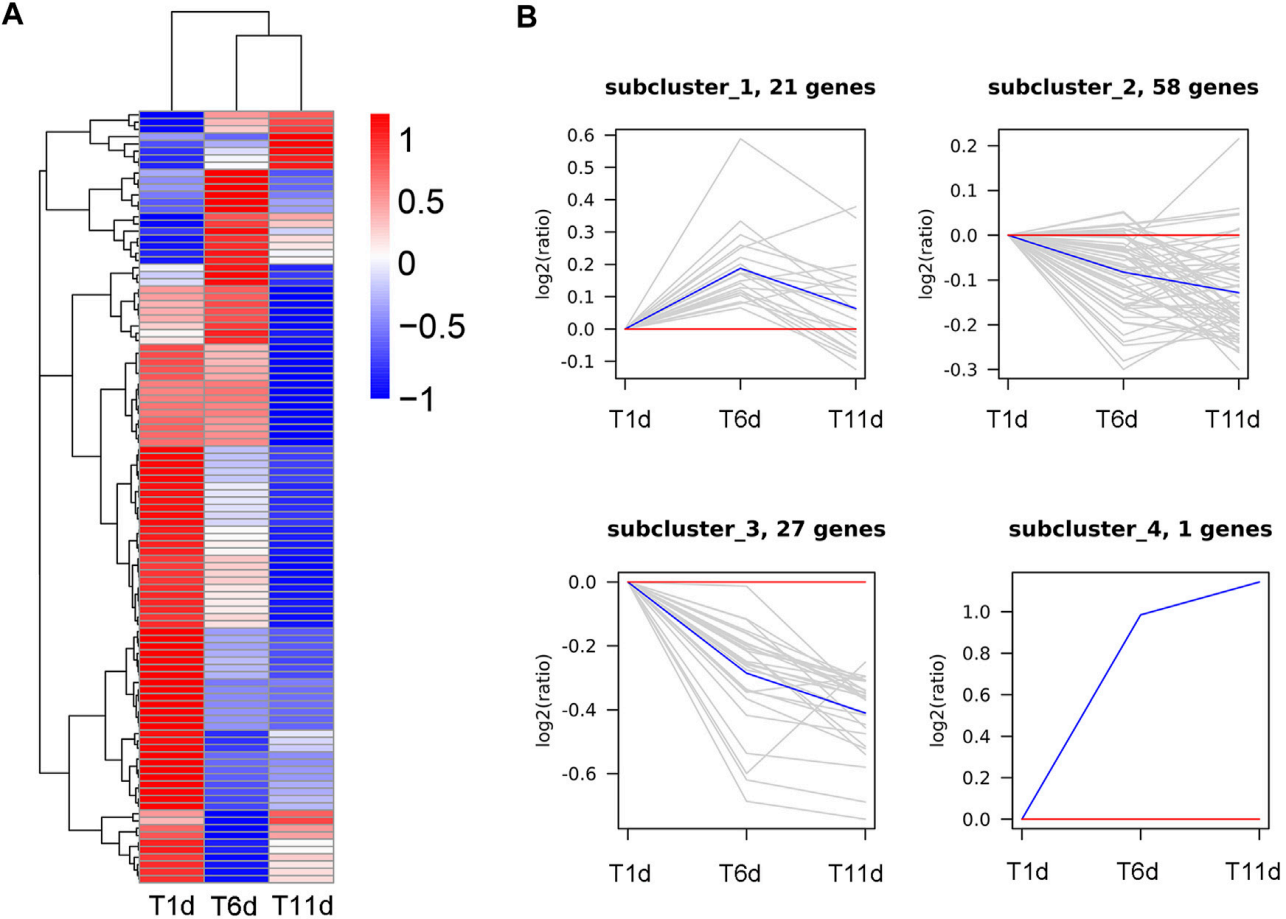
Clustering analysis of 107 *Drosophila* spermatogenesis-related orthologous genes throughout the testis development of *Z. tau*. **(A)** Heatmap of 107 orthologous genes on the basis of the average log2 (FPKM +1) values of three replicates. **(B)** Four sub-clusters of 107 orthologous genes by the H-means method. The blue line shows the average relative expression levels of all the genes in each subcluster, and the gray lines represent the relative gene expression levels of each gene in each subcluster.

In order to further explore the genes involved in spermatogenesis in *Z. tau*, the proteins involved in *Drosophila* spermatogenesis were searched in Uniport database. A total of 107 proteins were obtained, and these proteins were aligned to the transcriptome database of the *Z. tau,* and finally, 107 orthologous genes were obtained (Supplementary Table S2). These 107 orthologous genes were clustered into 4 clusters using the H-means method on the basis of the average log2 (FPKM +1) values of all genes (Figure 7A). The cluster results showed that 21 genes were first upregulated and then downregulated; 58 genes showed a minor change in expression level; 27 genes showed a downregulated expression; and only one gene (innexin 2 (*inx2*)) was significantly upregulated (Figure 7B).

### *Rdh12*-like*, pdxk, rgn, and inx2 genes are required for male reproduction*

**FIGURE 8:**
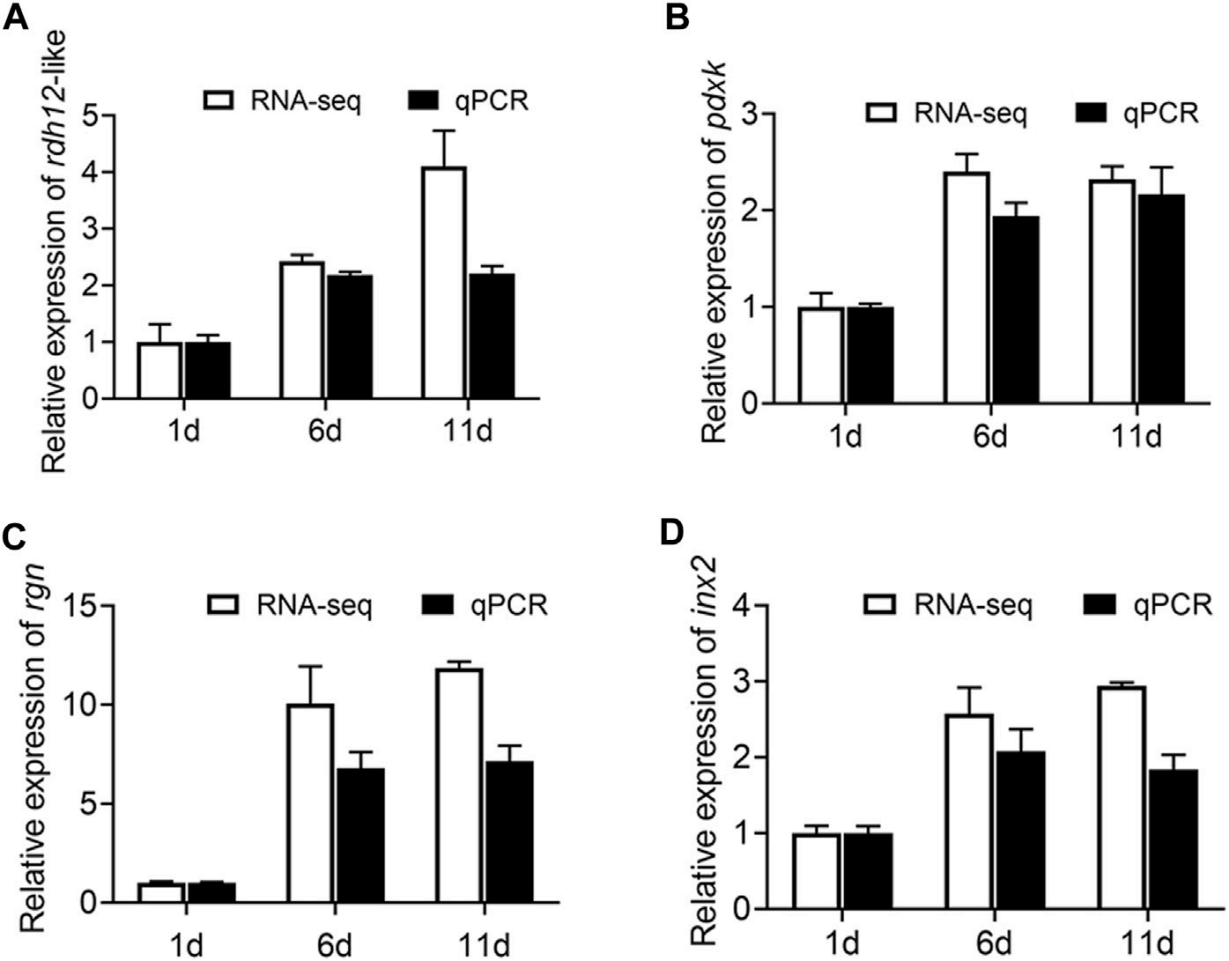
Expression of *rdh12*-like*, pdxk*, *rgn*, and *inx2* at three testis development stages (testis on day 1, 6, and 11 after eclosion). **(A–C)** Expression of 3 vitamin metabolism-related genes *rdh12*-like **(A)** *, pdxk* **(B)**, and *rgn* **(C)**. **(D)** Expression of *Drosophila* spermatogenesis-related orthologous gene *inx2* in *Z. tau.* In histogram, the black columns indicate the gene expression (FPKM), while the white columns denote the results of real-time PCR.

First, qRT-PCR was performed on these four genes (*rdh12*-like, *pdxk*, *rgn*, and *inx2*) to verify the accuracy of the RNA-Seq results. The results of RNA-Seq and qRT-PCR exhibited a high-level consistency, indicating that the RNA-Seq data were reliable and accurate (Figure 8).

**FIGURE 9:**
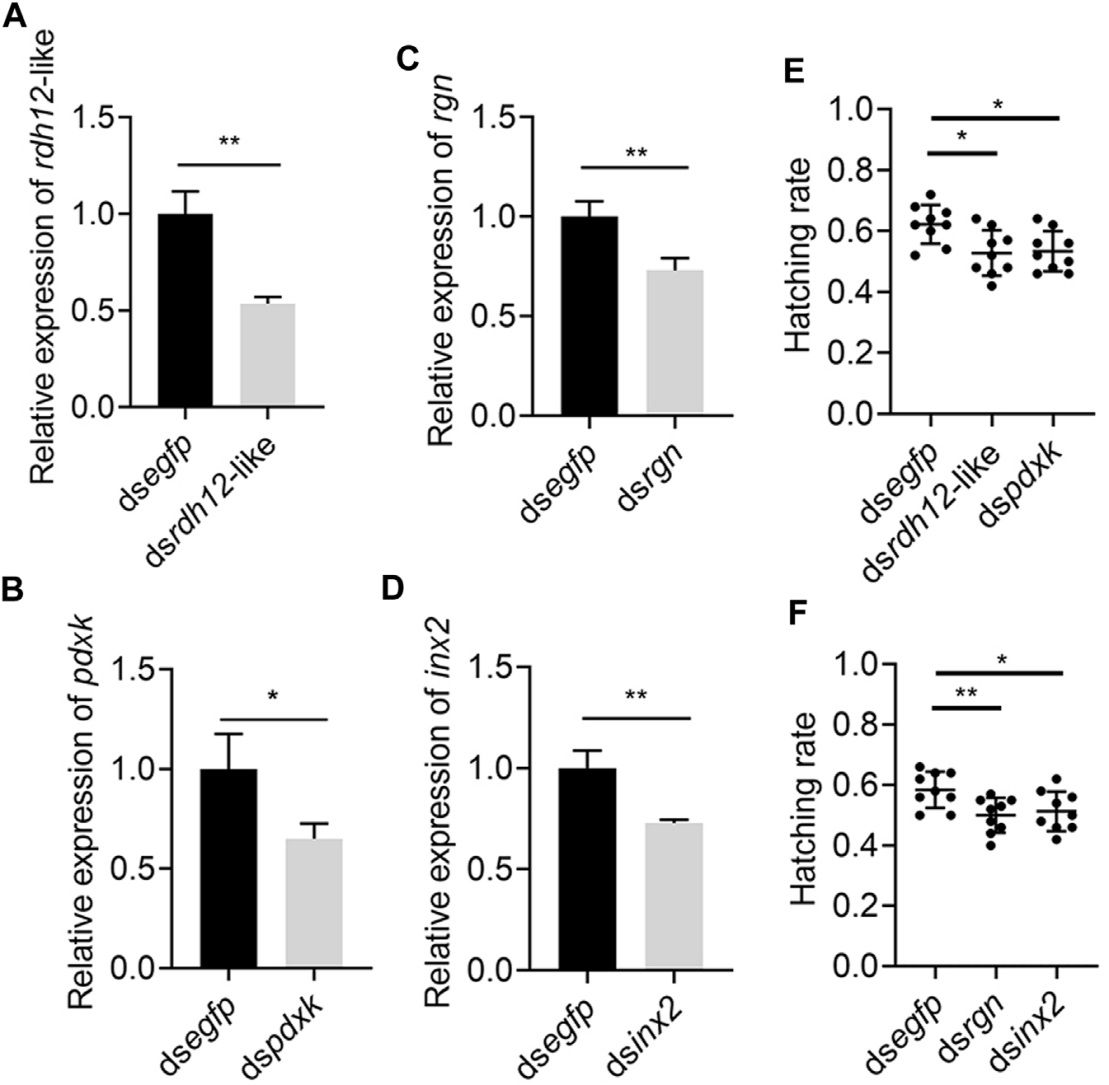
Knockdown of *rdh12*-like, *pdxk*, *rgn,* and *inx2* reduces offspring hatching rate in *Z. tau*. The expression of 3 vitamin metabolism-related genes *rdh12*-like **(A)**, *pdxk* **(B)**, *rgn* **(C)**, and *Drosophila* spermatogenesis-related orthologous gene *inx2* **(D)** after RNA interference. Offspring hatching rate after injection with ds*rdh12*-like **(E)**, ds*pdxk* **(E)**, ds*rgn* **(F)** *,* and ds*inx2* **(F)**, respectively. (*, *p* < 0.05; **, *p* < 0.01).

The 4 genes (*Rdh12*-like*, pdxk, rgn, and inx2*) were used for RNA interference to determine their function. The expression levels of *Rdh12*-like, *pdxk*, *rgn,* and *inx2* in the testes were decreased by 46.48%, 34.94%, 26.93% and 27.05% at 48 h after injection of the target gene dsRNA, respectively (*p* = 0.0028, *p* = 0.0343, *p* = 0.0091, and *p* = 0.0060) (Figures 9A–D). In addition, the RNAi-treated individuals were mated and exhibited a lower hatching rate in the four treatment groups than in the control group (*p* = 0.0108, *p* = 0.0104, *p* = 0.0077, and *p* = 0.0287) (Figures 9E, F). These results indicated that these four genes were necessary for male reproduction. Phylogenetic analysis showed that *rdh12*-like, *pdxk*, *rgn*, and *inx2* were highly conserved in tephritid species, and they were phylogenetically close to those in *Z. cucurbitae* (Supplementary Figure S2). Ka/Ks analysis results showed that the Ka/Ks value of four genes were much lower than 1, showing strong purifying selection (Supplementary Table S3).

## Discussion

In this study, the transcriptome of testes in different developmental stages were subjected to Iso-Seq and RNA-Seq. The analysis results of DEGs related to testis development indicated that the DEGs were mainly from the comparison of T11 vs. T1 and T6 vs. T1, rather than T11 vs. T6, indicating that the metabolic activity of the testis was stronger in the early stage (day 1–6 post eclosion), which was consistent with the change trend in testicular morphology at three time points. Furthermore, we found that retinol metabolism-, vitamin B6 metabolism-, and ascorbate and aldarate metabolism-related genes were involved in testis development. Previous studies have shown that male fertility depends on the balance between process of cell proliferation, cell differentiation and apoptosis, and this balance must be maintained throughout the life cycle. Vitamins play an important role in this balance (Bowles et al., 2006; Kim and Soh, 2009). Vitamins, as nutritional modulators, can reduce oxidative stress and regulate sperm mitochondrial function, and for example, specific vitamin supplementation can increase sperm quality in rats, demonstrating that vitamins contribute to male fertility (Dawson et al., 1992; Galatioto et al., 2007; Jensen et al., 2011; Shum et al., 2022).

Vitamin A, retinoic acid (RA), and retinol have been reported to be correlated with male reproduction (Clagett-Dame and Knutson, 2011). Vitamin A deficiency damages the seminiferous epithelium of the epididymis, prostate, and the seminal vesicle, thus resulting in the termination of spermatogenesis (Clagett-Dame and Knutson, 2011; Chihara et al., 2013). In dairy bulls, low-vitamin A diet decreases testis weight, daily sperm production, and epididymal sperm reserves (Rode et al., 1995). In rams, vitamin A deficiency causes poor semen quality and consequent low fertility (Abdulkareema et al., 2005). Retinoic acid, a vitamin A metabolite, plays an important role in all the stages of spermatogenesis, especially in the early stage of spermatogonia differentiation and spermatocyte meiosis (Clagett-Dame and DeLuca, 2002; Hogarth and Griswold, 2013). RA is synthesized from an inactive precursor through a two-step enzymatic oxidation reaction in which retinol is first converted into retinal then into RA. The first step is mediated by two enzyme families, namely, the cytosolic alcohol dehydrogenase (ADH) family, which is medium-chain dehydrogenases/reductases, and RDH family, which is short-chain dehydrogenases/reductases (Parés et al., 2008; Tong et al., 2012). In our study, knockdown the *rdh12*-like gene of RDH family reduced the hatching rate of *Z. tau* offspring. The result suggested that *rdh12*-like was essential for the male fecundity. Previous studies have shown that RDH10 is critical for RA biosynthesis (Niederreither et al., 1999; Sandell et al., 2007; Duester, 2008; Rhinn et al., 2011; Sandell et al., 2012; Tong et al., 2012). In juvenile mice, the deficiency of RDH10 in both Sertoli and germ cells results in a blockage of spermatogonial differentiation, and similar observation has been reported in vitamin A-deficient animals (Tong et al., 2012).

Pyridoxal 5′-phosphate (PLP) is the active form of vitamin B6, and it acts as a cofactor in over 140 different enzymatic reactions. The regulation of PLP biosynthesis and PLP homeostasis are important for the study of vitamin B6 nutrition. Unlike bacteria, the majority of organisms are not able to synthesize PLP, instead, they recycle it by inter-conversion of B6 vitamers such as pyridoxine (PN), pyridoxamine (PM), and pyridoxal (PL) present in the food, as a remedy. Pyridoxal kinase (PDXK) and pyridoxine/pyridoxamine 5′-phosphate oxidase (PNPO) cooperate to produce PLP. PDXK phosphorylates pyridoxine, pyridoxamine, and pyridoxal to produce PNP, PMP, and PLP, whereas PNPO oxidizes PNP and PMP into PLP (Mascolo et al., 2019). In the present study, knockdown the *pdxk* gene reduced the hatching rate of *Z. tau* offspring. This might be because the low expression of *pdxk* gene resulted in decreased PLP concentration, in turn leading to a significant decline in male fecundity. Previous studies have shown that *pdxk* is a gene highly expressed in adult human testis and spermatozoa, and this gene may play an important role in spermatogenesis and be related to male infertility. In *Drosophila*, a slight reduction in PLP level can compromise glucose homeostasis and DNA integrity, which are important for sperm production (Merigliano et al., 2018; Mascolo et al., 2019).

Vitamin C, also known as ascorbic acid, has various physiological functions including aiding in tissue development, acting as a co-factor of enzymes, and reducing DNA oxidative damage as a powerful antioxidant (Luck et al., 1995; Arrigoni and De Tullio, 2002; Vanderhout et al., 2021). Seminal ascorbic acid concentration can reach up to 10 times that of serum ascorbic acid (Charlesworth, 1991; Agarwal and Sekhon, 2010). The high-concentration ascorbic acid is the principal antioxidant in seminal plasma of fertile men, which can promote the development and reproduction of sperms and minimize their structural and functional defects (Luck et al., 1995; Lewis et al., 1997; Agarwal et al., 2004). Multiple studies have supported a positive association between vitamin C intake and serum concentration, semen ascorbic acid concentration, healthy semen parameters (including semen volume, and sperm concentration, number, motility, and morphology), or overall fertility (Yuan et al., 2013; Salas-Huetos et al., 2017). High dietary vitamin C intake has been linked to reduced sperm DNA damage, and low-level seminal vitamin C has been associated with high-level sperm DNA fragmentation in infertile men (Fraga et al., 1991; Song et al., 2006; Schmid et al., 2012; Rahimlou et al., 2019). Despite the evidence for beneficial effects of vitamin C on male fertility, an excessive intake of vitamin C poses the risk of oxidative stress since vitamin C is a pro-oxidant (Du et al., 2012). Further research is needed to determine vitamin C concentrations at which the oxidative stress is induced. *Rgn*, as a gluconolactonase, plays an important role in the biosynthesis of ascorbic acid (Kondo et al., 2006; Laurentino et al., 2011b). Our results showed that *rgn* knockdown reduced offspring hatching rate of *Z. tau*. In studies on rat and human, immunohistochemistry analysis indicated that RGN protein was broadly expressed in all testicular cell types, and the testicular localization of RGN protein suggested that it might exert important physiological functions in testis (Laurentino et al., 2011a). In addition, RGN also plays a role in suppressing oxidative stress (Son et al., 2006; Handa et al., 2009). The oxidative stress has been reported to cause defective spermatogenesis by damaging spermatogenic cells and sperm functions, which is one of the major causes of male infertility (Aitken and Curry, 2011). Moreover, RGN is also a calcium (Ca2+)-binding protein, playing an important role in regulating Ca2+ homeostasis, and the Ca2+ homeostasis disruption may cause reversible male infertility (Sengupta et al., 2007; Triphan et al., 2007).

In animals, the gonads contained two cell types, namely, the germ cell which develops into gametes and the soma which gives rise to other tissues (Fairchild et al., 2015). Importantly, gametogenesis requires ongoing cooperation between the germ cell and soma and disruption of somatic support cells can prevent the production of gametes and lead to sterility. Therefore, communication between the soma and germ line is essential for gametogenesis (Schedl, 1991). Previous studies have suggested a role of gap junction components in regulating soma-germline communication (Starich et al., 2014). When gap junction between the soma and germline is disrupted, germline differentiation is blocked, and germline stem cells are not maintained (Smendziuk et al., 2015; Huynh et al., 2022). Gap junctions are composed of innexin proteins in invertebrates (Stebbings et al., 2002; Bauer et al., 2005; Beyer and Berthoud, 2018). In the present study, *inx2* knockdown led to lower hatching rate, which might be attributed to the *inx2* knockdown-induced communication blockage between soma and germlines. Similar results have been reported in *Drosophila*. In the female *Drosophila*, injection of *inx-2* antibody results in follicle cell differentiation, nurse cell regression, oocyte growth, and choriogenesis defects (Bohrmann and Zimmermann, 2008). In the male *Drosophila*, soma-germline interactions play essential roles in regulating cell proliferation, differentiation, and homeostasis in the gonad. Histological analysis reveals that the knockdown of *inx2* in soma results in small and rudimentary testes, or even sterility (Smendziuk et al., 2015). Our amino acid sequence alignment results showed that the similarity of the *inx2* gene in *D*. *melanogaster* and *Z. tau* was as high as 90.74%. We speculated that the function of *inx2* might be conserved, and that this gene might have the same action mechanism in *Z. tau* just as in *D*. *melanogaster*. Namely, gene *inx2* regulates spermatogenesis by participating in the communication between somatic cells and germ cells. However, the mechanism by which the *inx2* gene affects communication between the soma and germline requires further study.

Phylogenetic tree showed that these four genes were conserved in Tephritidae, suggesting that their functions might be similar in Tephritidae. The selection analysis results showed that the Ka/Ks values of these four genes were far lower than 1, indicating no positive selection. Little change in these genes during the evolution process contributed to the exploration of species origin. In this study, we found that interference with *rdh12*-like, *pdxk*, *rgn* and *inx2* caused no abnormal testicular development. This may be because the interference was performed on day 7 post emergence, and at this time point, the testis was close to maturity. We also found that interference with these four genes significantly reduced the hatching rate without affecting male mating ability, and thus these 4 genes had the potential to be targets of SIT. It should be noted that interference with these genes only reduced the hatching rate rather than completely sterilizing *Z. tau* males. Hence, in order to obtain lower-fertility males, it is suggested to combine the technology of interfering with the target genes with low-dose radiation to induce male sterility. Such a combination can further lower fertility of males whose target genes are interfered with, with their mating competitiveness unaffected, at low doses of radiation (Dyck et al., 2021). However, appropriate radiation dose remains to be further explored. In addition, *rdh12*-like, *pdxk*, *rgn* and *inx2* can also be used as gene drive targets. The low offspring hatching rate reduces the diffusion capacity of the *Z. tau* population without causing the population extinction. Future studies are suggested to investigate the long-term stability of gene drive so as to enhance the safety of SIT.

## Data Availability

The datasets presented in this study can be found in online repositories. The names of the repository/repositories and accession number(s) can be found below: https://www.ncbi.nlm.nih.gov/, SRR27009454 https://www.ncbi.nlm.nih.gov/, SRR27009453 https://www.ncbi.nlm.nih.gov/, SRR27009452 https://www.ncbi.nlm.nih.gov/, SRR27009451 https://www.ncbi.nlm.nih.gov/, SRR27009450 https://www.ncbi.nlm.nih.gov/, SRR27009449 https://www.ncbi.nlm.nih.gov/, SRR27009448 https://www.ncbi.nlm.nih.gov/, SRR27009447 https://www.ncbi.nlm.nih.gov/, SRR27009446 https://www.ncbi.nlm.nih.gov/, SRR27024225.

## Supplementary material

SUPPLEMENTARY FIGURE S1 Upset Plot of functional annotation of the new transcripts. The solid dots indicate that new transcripts were annotated to the corresponding database on the left, and the numbers on the top of bars denote the number of new transcripts annotated to one or more databases.

SUPPLEMENTARY FIGURE S2 Phylogenetic tree of RDH12-like **(A)**, PDXK **(B)**, RGN **(C)**, and INX2 **(D)** using MEGA7.0.26 by maximum likelihood method. The scale represents the distance. Numbers at each branch indicate bootstrap values based on 1000 replicates.

Supplementary Table 1 Primer sequences used in the present study.

Supplementary Table 2 Expression of orthologous genes associated with *Drosophila* spermatogenesis.

Supplementary Table 3 The Ka and Ks of *rdh12*-like, *pdxk*, *rgn*, and *inx2* genes.
