# Supplementary material for: Identification of testis development-related genes by combining Iso-Seq and RNA-Seq in Zeugodacus tau
Source: Front Cell Dev Biol. 2024 Mar 11;12:1356151. doi: 10.3389/fcell.2024.1356151 (PMC10961823; doi:10.3389/fcell.2024.1356151)
Supplement: Supplementary file 3 [file Table1.DOCX]

**Table S1** Primer sequences used in the present study

| **Primer name** | **Sequence (5’ – 3’)** |  |
| --- | --- | --- |
| **Primer for gene cloning** | |  |
| *rdh12*-like-F | | GCACAAAGCAAGAAACGAGC |
| *rdh12*-like-R | | CATTTCTTCAGAAAGACGCG |
| *pdxk*-F | | CGAAATGGCTAATCCAGACT |
| *pdxk*-R | | TAAGTTCGCACTCCTGTGCA |
| *rgn*-F | | ATCGAACCCATCCCTAATGG |
| *rgn*-R | | GTGTGATTACTGAGGCGCTT |
| *inx2*-F | | CCATAGCACCACCAAACTCT |
| *inx2*-R | | CCACTAAGCGGCGAATTGTT |
| **Primers for Quantitative Real-time PCR** | |  |
| q-*rdh12*-like-F | TTGTGGTGGTAAGTTCAGCG |  |
| q-*rdh12*-like-R | CAGCCAACTTTCGTGTGAAC |  |
| q-*pdxk*-F | ACTCTTCTTAGCGCACAGTT |  |
| q-*pdxk*-R | TAAGTTCGCACTCCTGTGCA |  |
| q-*rgn*-F | ACTCTGATTGGTGAAGGCCC |  |
| q-*rgn*-R | TCGCCTTCGACGGGTATAAT |  |
| q-*inx2*-F | TATTGATCGCCTTCTCGCTG |  |
| q-*inx2*-R | TTGTACAACATCATGGCCCG |  |
| *rpl32*-F | TTAGGTCTATTCGTTCTCCTGTG |  |
| *rpl32*-R | TTGGTCCACAATGTCCGTGA |  |
| **Primers for RNAi** | |  |
| ds-*rdh12*-F | GGATCCTAATACGACTCACTATAGGTCGTGACGGGCTGTAATACG |  |
| ds-*rdh12*-R | GGATCCTAATACGACTCACTATAGGGACCCAAGTGATTCACACCA |  |
| ds-*pdxk*-F | GGATCCTAATACGACTCACTATAGGTGTGATCCTGTCATGGGAGA |  |
| ds-*pdxk*-R | GGATCCTAATACGACTCACTATAGGTGATATGCCGTTTCCTTGGC |  |
| ds-*rgn*-F | GGATCCTAATACGACTCACTATAGGCGGTCGTCGTGTAGTTGTGA |  |
| ds-*rgn*-R | GGATCCTAATACGACTCACTATAGGGCCACACCGGTTTCAACATT |  |
| ds-*inx2*-F | GGATCCTAATACGACTCACTATAGGCGGTGTGGACGAGGTGAAAT |  |
| ds-*inx2*-R | GGATCCTAATACGACTCACTATAGGCCATCGGATCGATACGTTCA |  |
| *dsegfp*-F | GGATCCTAATACGACTCACTATAGGACGTAAACGGCCACAAGTTC |  |
| *dsegfp*-R | GGATCCTAATACGACTCACTATAGGAAGTCGTGCTGCTTCATGTG |  |
